# Yishen Gushu formula exerts osteoprotective effects in OVX-Induced PMOP rats: role of ferroptosis Inhibition and iron metabolism correction

**DOI:** 10.1186/s41065-026-00642-5

**Published:** 2026-02-04

**Authors:** Bo Jin, Hao Zeng, ZhiFeng Wei, YongJin Li, XiaoYun Zhang

**Affiliations:** 1 Guangxi University of Chinese Medicine, Nanning, Guangxi 530200 China; 2 Ruikang Hospital Affiliated to Guangxi University of Chinese Medicine, No. 10 Huadong Road, Nanning City, Guangxi Zhuang Autonomous Region 530011 China

**Keywords:** YSGSF, PMOP, Bone metabolism, Oxidative stress, NCOA4/GPX4, Ferroptosis

## Abstract

**Background:**

Postmenopausal osteoporosis (PMOP) is a common bone disease among middle-aged and elderly women. Ferroptosis, a form of programmed cell death, plays a crucial role in the regulation of bone metabolism. Yishen Gushu Formula (YSGSF), a traditional Chinese medicine (TCM) compound, has clinical potential for treating PMOP, yet its specific mechanism of action remains unclear.

**Objective:**

This study aims to investigate the specific mechanism by which YSGSF treats PMOP by inhibiting ferroptosis through mediating the NCOA4/GPX4 pathway.

**Methods:**

The rat PMOP model was established by OVX and treated with YSGSF for 12 consecutive weeks. HE staining and micro-CT were used to examine bone tissue morphology and structure. Serum levels of E2, PINP, and β-CTX were measured by ELISA. RNA-seq analysis was performed to explore the potential mechanism of YSGSF. Oxidative stress markers (ROS, MDA, SOD, GSH, FE) in rat serum were determined. The regulatory effects of YSGSF on NCOA4, FTH1, GPX4, and SLC7A11 were evaluated by IHC and qRT-PCR. Erastin was used in vitro to induce ferroptosis in ROS17/28 osteoblasts, with cell viability and ferroptosis-related protein levels detected by CCK8 and Western blotting.

**Results:**

YSGSF improved bone metabolism in OVX rats, alleviated osteoporotic morphological changes, promoted the overall structural recovery of trabecular bone, and increased bone density. RNA-seq suggested that YSGSF may exert its therapeutic effect on PMOP by inhibiting ferroptosis. Subsequent results indicated that YSGSF improved iron metabolism in bone tissue and suppressed iron-dependent oxidative damage. It reduced serum iron ion accumulation and inhibited the production of ROS and MDA. IHC and qRT-PCR confirmed that YSGSF suppressed the expression of NCOA4, upregulated FTH1, and further increased the expression levels of GPX4 and SLC7A11. In vitro cell experiments demonstrated that YSGSF ameliorates Erastin-induced ferroptosis in osteoblasts, improving the expression of related proteins and cell viability.

**Conclusion:**

YSGSF ameliorates OVX-induced PMOP, potentially by inhibiting osteoblast ferroptosis through modulation of the NCOA4/GPX4 pathway.

**Supplementary Information:**

The online version contains supplementary material available at 10.1186/s41065-026-00642-5.

## Introduction

Postmenopausal osteoporosis (PMOP) is characterized by decreased bone mineral density, deteriorated bone microstructure, and increased fracture risk, and it primarily affects women with estrogen deficiency due to menopause [1]. As a common metabolic bone disease, PMOP is usually asymptomatic before the onset of fragile fractures [2]. Approximately 30% of postmenopausal women over 50 years old worldwide are affected by this condition, and the associated medical costs have surged, highlighting the public health urgency of this issue [3]. The pathogenesis of PMOP involves multiple factors, including hormones, genetics, and environment. Although progress has been made in understanding the pathophysiology of PMOP, challenges remain in its early diagnosis and treatment. Currently, the main intervention methods are still calcium supplementation, vitamin D supplementation, and estrogen replacement therapy (ERT); however, their clinical application has significant limitations. Long-term use of estrogen significantly increases the risk of endometrial cancer and breast cancer [4], while the gastrointestinal adverse reactions of bisphosphonates lead to insufficient patient compliance [5, 6]. These limitations underscore the urgency of developing therapeutic strategies with both safety and multi-target regulatory efficacy.

Traditional Chinese Medicine (TCM), especially TCM compound prescriptions, has emerged as a promising therapeutic strategy in recent years due to its multi-targeted and holistic regulatory effects, as well as fewer adverse reactions [7, 8]. The pharmacological mechanism of TCM in preventing and treating PMOP is often consistent with the “kidney governing bone” theory; therefore, the selected Chinese herbal medicines mostly possess the efficacy of tonifying the kidney and replenishing essence. For instance, classic TCM compound prescriptions such as Erxian Decoction (EXD) [9] and Zuogui Pill (ZGP) [10] are commonly used clinically for treating PMOP patients with kidney deficiency and blood depletion syndrome. Gusuibu (Rhizoma Drynariae) improves the bone microenvironment by inhibiting oxidative stress [11]. Fufang Lurong Jiangu capsule (FLJC) can enhance the antioxidant capacity of osteoblasts by activating the Nrf2/HO-1 pathway [12]. Qing`e Pill (QEP) improves cell activity by inhibiting ferroptosis and reverses bone loss in ovariectomized (OVX) rats [13]. Most kidney-tonifying herbs have been confirmed to regulate bone metabolism by modulating mechanisms such as immunity, oxidative stress, and ferroptosis [14–18].

Ferroptosis is a type of cell death modality driven by iron-dependent lipid peroxidation, and its association with PMOP has attracted considerable attention in recent years [19, 20]. Emerging evidence indicates that oxidative stress and iron metabolism disorder, which interact through the ferroptosis and ferritinophagy pathways, are key mechanisms driving bone deterioration [21–24]. Studies have indicated that postmenopausal estrogen deficiency downregulates GPX4, leading to redox imbalance in osteoblasts [17]. On the other hand, low estrogen levels increase ferritin export via FPN1, resulting in ferritin overload; this then generates reactive oxygen species (ROS) through the Fenton reaction, providing substrates for the occurrence of ferroptosis [25]. Nclear receptor coactivator 4 (NCOA4)-mediated ferritinophagy degrades the ferritin heavy chain 1 (FTH1) and ferritin light chain (FTL) subunits, releasing labile iron ions and further exacerbating oxidative stress [26, 27]. In PMOP, elevated NCOA4 has been shown to be closely associated with increased cytoplasmic labile iron ions and lipid peroxidation, a phenomenon observed in both aged ovarian and bone tissues [25, 28]. Notably, the imbalance of the NCOA4-FTH1 axis not only directly triggers the ferroptosis process [29] but also synergistically inhibits the glutathione peroxidase 4 (GPX4)/solute carrier family 7 member 11 (SLC7A11) system system by enhancing the sensitivity to lipid peroxidation [30], reduces SOD activity, and impairs the cellular antioxidant defense capacity [31–35], thus forming a cascade amplification effect and ultimately leading to bone tissue damage [36].

Yishen Gushu Formula (YSGSF) was developed by Professor Huang Yourong, a renowned senior TCM physician from Guangxi, China. In this formula, *Epimedii Folium* (Yinyanghuo) and *Rehmanniae Radix Praeparata* (Shudihuang) serve as the sovereign herbs. They are accompanied by kidney-tonifying and bone-strengthening herbs such as *Rhizoma Drynariae* (Gusuibu) and Cortex *Eucommiae Praeparata* (Yanduzhong). Additionally, *Radix Ginseng* (Renshen) and *Astragali Radix* (Huangqi) act as auxiliary herbs to invigorate the spleen and replenish qi, while *Rhizoma Chuanxiong* (Chuanxiong) and *Radix Aucklandiae* (Muxiang) soothe the liver and promote qi circulation, and *Panax notoginseng* (Sanqi) activates blood circulation and dredges collaterals. Collectively, these herbs exert the synergistic effects of tonifying the kidney, invigorating the spleen, activating blood circulation, and strengthening bones, embodying the TCM concept of “syndrome differentiation and treatment” based on theories of “liver and kidney sharing the same origin” and “spleen and stomach nourishing each other.”

Previous clinical studies have confirmed that YSGSF can significantly improve patients’ bone mineral density (BMD) by regulating the bone formation-resorption coupling mechanism. Furthermore, network pharmacology predictions combined with animal experiments have revealed that its anti-inflammatory effect may be associated with downregulating the expression of TNF-α, IL-1β, and c-Jun [37]. In addition, 84 active components in YSGSF were successfully identified in previous studies, and the optimal drug concentration was determined. The present study further confirmed via RNA-seq that the therapeutic effect of YSGSF on PMOP may be linked to mechanisms such as iron metabolism and ferroptosis. Based on this, this study aims to verify whether YSGSF can improve ferroptosis-induced PMOP by regulating the NCOA4/GPX4 signaling pathway. Through further mechanistic investigations, this study intends to provide an intervention strategy for PMOP treatment that differs from conventional approaches, which holds certain scientific value and potential for clinical transformation.

## Materials and Methods

### Experimental animals

A total of 24 specific pathogen-free (SPF) female Sprague-Dawley (SD) rats, aged 12 weeks and weighing 260 ± 20 g, were purchased from Hunan Slack Jingda Laboratory Animal Co., Ltd., China, with a quality certificate number of 430,727,241,100,893,758. The rats were housed in the barrier system of the SPF Laboratory Animal Center at Guangxi University of Chinese Medicine, China. The husbandry conditions were maintained as follows: room temperature of 22 ± 3 °C, relative humidity of 40%–70%, and a 12 h light/12 h dark cycle. Throughout the experiment, the rats had ad libitum access to standard diet and sterile drinking water.

This animal experimental protocol was reviewed and approved by the Laboratory Animal Ethics Committee of Guangxi University of Chinese Medicine, with an approval number of DW20231109-234. the entire experiment was conducted in strict adherence to the Regulations on the Administration of Laboratory Animals and relevant ethical standards.

### Experimental drugs

The specific composition and names of the experimental drug Yishen Gushu Formula (YSGSF) are presented in Table 1. All herbal materials were subjected to comprehensive inspection by the Quality Inspection Center of Guangxi Xianzhu Traditional Chinese Medicine Technology Co., Ltd., China, in accordance with the standards specified in the *Pharmacopoeia of the People’s Republic of China (2020 Edition)*, and complied with the quality standards for TCM decoction pieces.

The control drug used was alendronate sodium tablets (70 mg per tablet, batch number Y011893), manufactured by Merck Sharp & Dohme (Hangzhou) Pharmaceutical Co., Ltd., China.

**Table 1:** Composition of YSGSF

Latin name	Chinese name	Weight(g)	Batch number
*‌Epimedii Folium*	Yinyanghuoye	30	20,240,302
*Rehmanniae Radix Praeparata*	Shudihuang	30	20,240,201
*‌Rhizoma Drynariae*	Gusuibu	15	20,240,102
*Cortex Eucommiae Praeparata*	Yanduzhong	15	20,240,301
*Cuscutae Semen Praeparatum*	Yantusizi	15	20,240,301
*Radix Ginseng*	Renshen	10	20,240,501
*‌Astragali Radix*	Huangqi	20	20,240,403
*Rhizoma Chuanxiong‌*	Chuanxiong	6	20,240,202
*Radix Aucklandiae*	Muxiang	6	20,240,501
*Panax notoginseng*	Sanqi	6	20,240,301
Total amount		153	

### Experimental reagents

The Rat Serum PINP Detection Kit (Cat. No.: MM-20710R1) and Rat Serum β-CTX Detection Kit (Cat. No.: MM-20172R1) were purchased from Jiangsu Enzyme-Linked Immunosorbent Assay (ELISA) Industry Co., Ltd., China. 4% paraformaldehyde (Cat. No.: G1101) and EDTA Decalcifying Solution (Cat. No.: G1105) were obtained from Wuhan Servicebio Technology Co., Ltd., China. NCOA4 Antibody (Cat. No.: A04368-3) and Anti-xCT/SLC7A11 Antibody (Cat. No.: A03036-2) were purchased from Wuhan Boster Biological Technology Co., Ltd., China. Anti-Glutathione Peroxidase 4 Rabbit Monoclonal Antibody (Cat. No.: A11243) and Anti-Ferritin Heavy Chain Rabbit Monoclonal Antibody (Cat. No.: A19544) were acquired from Wuhan Abcam Technology Co., Ltd., China. The Competitive Quantitative ELISA Kit for Glutathione (Cat. No.: EEL155), Competitive Quantitative ELISA Kit for Malondialdehyde (Cat. No.: EEL160), and Quantitative ELISA Kit for Rat Ferritin (Cat. No.: EEL129) were purchased from Thermo Fisher Scientific Inc., USA. The Total RNA Extraction Kit (containing chloroform substitute, Cat. No.: R1201) and TaqMan One-Step RT-qPCR Kit (Cat. No.: T2210) were obtained from Beijing Solarbio Science & Technology Co., Ltd., China. Rat osteoblast cell line ROS1728 (Catalog No.: YS3036C) was purchased from Shanghai YaJi Biological Technology Co., Ltd. Erastin (Cat. No.: IE0310) was purchased from Beijing Solarbio Science & Technology Co., Ltd. The CCK-8 high-sensitivity rapid detection kit (Catalog No.: CR2411091) was purchased from Wuhan Servicebio Technology Co., Ltd.

### Preparation of drug solutions

#### YSGSF solution Preparation

A total of 153 g of YSGSF herbal materials were weighed, soaked in 10-fold volume of ultrapure water at room temperature for 20 min, boiled at 100℃, then decocted at 85℃ for 1 h, and initially filtered through a 200-mesh nylon filter cloth. The herbal residue was re-decocted with 8-fold volume of ultrapure water for 40 min. The combined filtrates were concentrated to a final concentration of 1.944 g/mL (herb equivalent per mL), aliquoted into sterile centrifuge tubes, and stored at 4℃.

Alendronate sodium tablet powder was weighed, added to ultrapure water preheated to 60℃, and ultrasonically dispersed for 30 min. After standing for 30 min, the supernatant was collected, and the final concentration was adjusted to 0.32 g/mL. All drug solutions were equilibrated at room temperature for 30 min before use.

The preparation methods described above were consistent with those in previous studies [37]. The drug concentration employed in this study (1.944 g/mL) was determined by converting clinical doses based on animal body surface area, and its efficacy and safety have been validated in previous studies.

### Establishment of PMOP model and experimental grouping

The postmenopausal osteoporosis model was established via bilateral ovariectomy (OVX). After 6 h of fasting, the rats were anesthetized via intraperitoneal injection of 3% sodium pentobarbital (30 mg/kg). A 1.5 cm longitudinal incision was made 2 cm below the costal margin; after bluntly dissecting the muscle layer, the ovarian fat pad was identified. The fallopian tubes and blood vessels were double-ligated with 6 − 0 silk suture, followed by complete removal of the ovaries. For the Sham group (sham-operated group), only an equivalent amount of adipose tissue was removed. Postoperatively, penicillin sodium (4 × 10⁴U/kg) was intramuscularly injected for 3 consecutive days to prevent infection. The construction method of the PMOP model used in this study refers to the previous research content [37].

Seven days after surgery, the rats were randomly divided into four groups (*n* = 6 per group): Sham group, OVX group, ALN group, and YSGSF group. The ALN group and YSGSF group were continuously administered the pre-prepared ALN suspension and YSGSF solution, respectively, for 12 weeks; the Sham group and OVX group were only given an equal volume of normal saline via gastric gavage. The gavage volume was calibrated daily based on the rats’ body weight, and body weight changes were recorded weekly.

### ELISA

After the final administration, rats were fasted for 12 h and blood was collected via abdominal aorta puncture. Whole blood samples were coagulated at 4℃ for 2 h, centrifuged at 3000 rpm for 15 min at 4℃, and the supernatant was aliquoted into EP tubes for storage at -80℃. Serum levels of PINP, β-CTX, GSH, MDA, ROS, and FE were measured using commercial ELISA kits according to the manufacturers’ protocols.

### Micro-CT

Right femoral samples (*n* = 3) were fixed in 4% paraformaldehyde for 48 h and scanned using a Micro-CT system under consistent background conditions. Scanning parameters were set as follows: voltage 80 kV, current 0.06 mA. Using the distal femoral growth plate as the anatomical landmark, the trabecular bone region 1 mm proximal to the growth plate (metaphyseal cancellous bone) was selected for parameter acquisition. After scanning, 3D reconstruction and quantitative analysis of bone microstructure were performed, including trabecular thickness (Tb.Th), trabecular number (Tb.N), trabecular separation (Tb.Sp), trabecular pattern factor (Tb.Pf), bone mineral density (BMD), and bone volume fraction (BV/TV).

### HE

Right femoral samples of rats (*n* = 3) were fixed in 4% paraformaldehyde for 48 h, then subsequently decalcified with EDTA decalcifying solution for 4 weeks—with fresh decalcifying solution replaced once a week during this period. After decalcification, paraffin embedding was performed, and serial sections with a thickness of 5 μm were finally prepared from the paraffin blocks. Following dewaxing in xylene, the sections were stained with hematoxylin and eosin in accordance with the instructions of the HE staining kit. Finally, neutral gum was dropped onto the sections for mounting. Pathological changes in the bone tissue were observed under a microscope, and the continuity of trabecular bone as well as changes in bone morphology were evaluated.

### RNA-Seq

Total RNA was extracted from the left femurs of OVX and YSGSF group rats (*n* = 6) using TRIzol reagent. RNA quality was assessed, and qualified samples were used for library construction. mRNA was purified from total RNA using oligo (dT) magnetic beads, followed by PCR amplification to generate the final cDNA library. Library quality was evaluated with an Agilent Bioanalyzer 4150. Sequencing was performed on the Illumina Novaseq 6000/MGISEQ-T7 platform. All procedures wereoperated by Shanghai Applied Protein Technology (APTBIO), China.

### RNA-Seq data analysis

Raw sequencing data were first filtered, and gene Count values were calculated based on transcript length. Differential expression analysis for biologically replicated samples was conducted using the DESeq2 R package, with significance thresholds set as |log2FC| > 1 and Q-value < 0.05. Protein-protein interaction (PPI) networks of differentially expressed genes (DEGs) were constructed via the STRING database (https://string-db.org/). Gene Ontology (GO) and Kyoto Encyclopedia of Genes and Genomes (KEGG) enrichment analyses were performed using the clusterProfiler R package, with *P* < 0.05 indicating significant enrichment.

### IHC

Paraffin-embedded femoral sections were dewaxed in xylene and hydrated through graded ethanol. Antigen retrieval was performed via high-pressure heat treatment (121℃, 10 min in 0.01 M citrate buffer, pH 6.0). Endogenous peroxidase activity was blocked with 3% H2O2 for 15 min at room temperature, followed by blocking with 5% bovine serum albumin (BSA). Sections were incubated with primary antibodies against NCOA4, FTH1, GPX4, and SLC7A11 (1:200 dilution) at 4℃ overnight. After PBS washing, secondary antibodies (1:500 dilution) were applied for 1 h at room temperature. Color development was performed using DAB, followed by hematoxylin counterstaining and mounting with neutral gum. Images were acquired under a microscope, and positive staining area (%Area) was quantified using ImageJ software.

### qRT-PCR

Femoral tissues were homogenized in liquid nitrogen and lysed with 1 mL RNA extraction reagent. After centrifugation (12,000 rpm, 10 min, 4℃), the supernatant was subjected to RNA purification using chloroform, isopropanol, and 75% ethanol. RNA concentration was adjusted to 500 ng/µL. Reverse transcription and PCR amplification were performed using the TaqMan One-Step RT-qPCR Kit. Relative mRNA expression of NCOA4, FTH1, GPX4, and SLC7A11 was calculated using the 2^−^ΔΔCt method, normalized to GAPDH. Primers (Table 2) were synthesized by Wuhan Servicebio Technology Co., Ltd., China.

**Table 2:** Primer sequences

Primer	Sequence(5’-3’)	Length(dp)
GAPDH	CTGGAGAAACCTGCCAAGTATG	138
	GGTGGAAGAATGGGAGTTGCT	
NCOA4	AAAGAAGGGAAGGACAAGAATGG	126
	GGGTGTCTTAGCGTGTTCTGTTA	
FTH1	CGCCAGAACTACCACCAGGACT	184
	TCAGTTTCTCAGCATGTTCCCTC	
GPX4	AGGCAGGAGCCAGGAAGTAATC	212
	ACCACGCAGCCGTTCTTATC	
SLC7A11	TATGCTGAATTGGGTACGAGC	114
	TATTACCAGCAGTTCCACCCA	

### CCK-8 assay for cell viability

To assess cell proliferation viability, the CCK-8 assay was employed. ROS17/28 osteoblasts in the logarithmic growth phase were seeded in 96-well plates at a density of 5 × 10³ cells/well. After 24 h of adherence, the cells were treated with 2.15 µM Erastin and serum-containing medium supplemented with 5% YSGSF (the concentration selection referred to Supplementary Material 1). The blank group contained only complete medium, the control group consisted of medium, cells, and solvent control DMSO (concentration less than 0.1%); the Erastin group included complete medium with 2.15 µM Erastin solvent and osteoblasts; the YSGSF group contained complete medium with 5% YSGSF drug-containing serum and 2.15 µM Erastin, along with cells. After the 48-hour intervention, 10 µL of CCK-8 reagent was added to each well and incubated in the incubator protected from light. The absorbance of each well was measured at 450 nm wavelength using a microplate reader. Cell viability was expressed as a percentage, calculated by the formula: (Experimental group - Blank group) / (Control group - Blank group) × 100%.

### Western blotting

Collect the treated ROS17/28 cells and extract total proteins using RIPA lysis buffer containing protease and phosphatase inhibitors. Measure protein concentration by BCA assay and load equal amounts of proteins. Separate proteins by molecular weight using SDS-PAGE, then transfer the proteins to PVDF membranes via wet transfer method. Block the membranes with 5% skim milk for 1 h, incubate with primary antibodies at 4℃ overnight, followed by incubation with horseradish peroxidase-conjugated secondary antibodies at room temperature the next day. Capture signals using an imaging system. Quantify band intensity using ImageJ software, and express relative protein levels as the ratio of target protein gray value to internal reference protein gray value.

### Statistical analysis

All experimental data were analyzed using GraphPad Prism 10 software. Quantitative data were expressed as mean ± standard deviation (x̄ ± s). If the data met the assumptions of normal distribution and homogeneity of variance, one-way analysis of variance (ANOVA) was used for intergroup comparisons. A P-value < 0.05 was considered statistically significant.

## Results

### YSGSF improves general conditions of OVX rats

As shown in Table 3, Week 0 refers to the pre-surgical period, during which the body weight of rats in each group was 260 ± 20 g, with no significant difference among groups. In the first week after surgery, there was still no significant difference in body weight across all groups, and all groups exhibited a steady weight gain; meanwhile, the ALN group and YSGSF group were administered the corresponding drugs.

In the second week after surgery, compared with the Sham group, the OVX group, ALN group, and YSGSF group all showed different degrees of weight gain. Among them, the OVX group exhibited the most rapid weight gain (*P* < 0.01), indicating that the sharp decline in estrogen levels after OVX led to rapid weight gain. In contrast, the drug-treated groups (ALN and YSGSF groups) showed a slower weight gain compared with the OVX group.

**Fig. 1:**
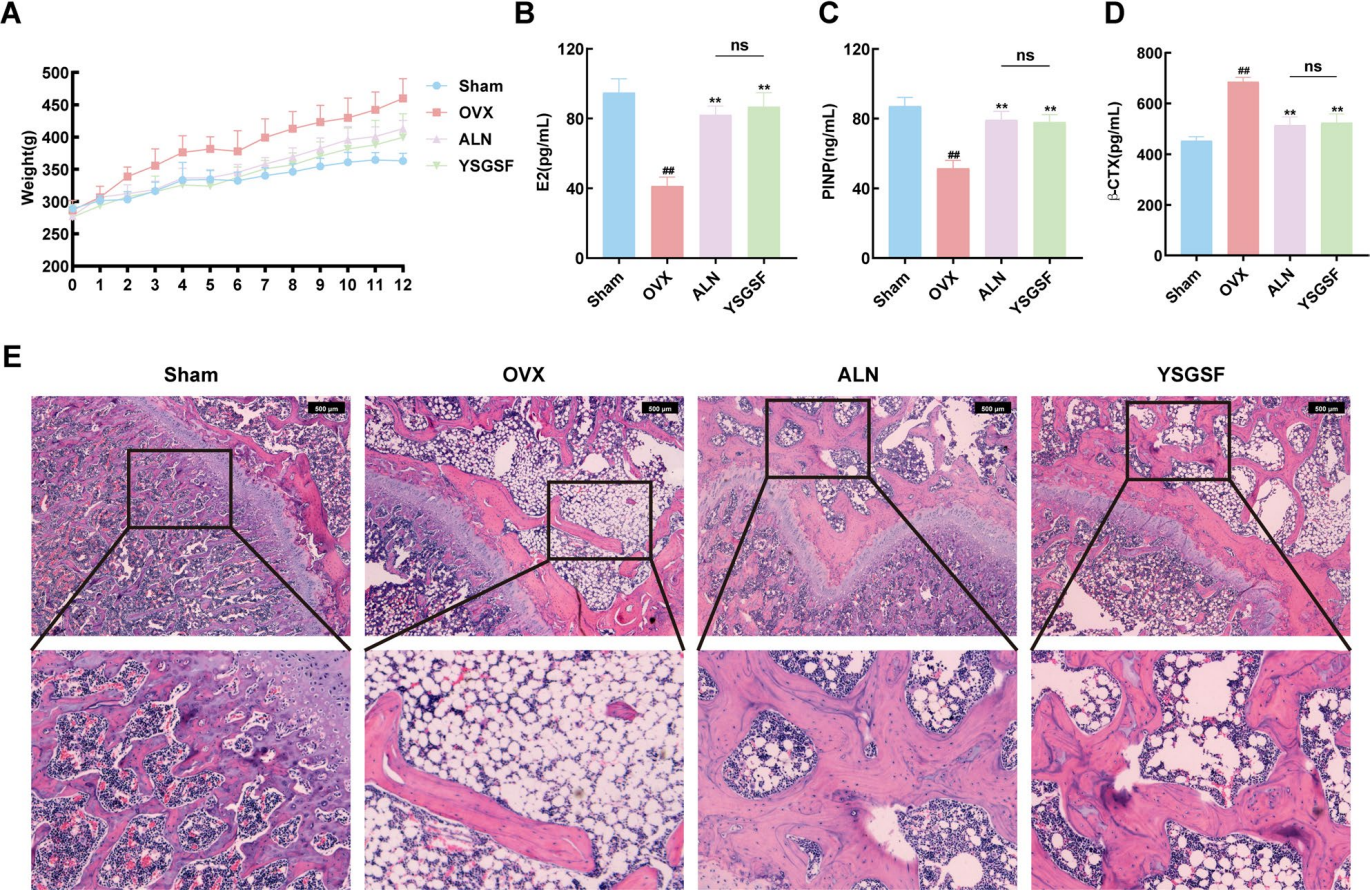
YSGSF improves the general condition and osteoporosis in OVX rats. **A** Body weight of rats in each group. **B** Serum E2 levels in each group. **C** Serum PINP levels in each group. **D** Serum β-CTX levels in each group. (*n*=6) (**E**) HE of the distal femur in each group; (*n*=3); Compared to the Sham group, #*p*<0.05, ##*p*<0.01; Compared to the OVX group, *p < 0.05, **p < 0.01; ^ns^ *p*＞0.05

In each subsequent week, the OVX group maintained a stable and rapid weight gain, and the body weight difference between the OVX group and the Sham group remained at 40 ± 10 g. Compared with the OVX group, the ALN group and YSGSF group showed a gentler growth trend. These results indicate that ALN and YSGSF treatments can alleviate the rapid weight gain caused by the sharp decline in estrogen. The specific growth trend is shown in Fig. 1A.

The serum E2 levels of each group are shown in Fig. 1B. Compared with the Sham group, the E2 level in the OVX group significantly decreased (*P* < 0.01), indicating the significant impact of OVX surgery on rat estrogen levels. Compared with the OVX group, both the ALN group and YSGSF group showed significantly increased E2 levels (*P* < 0.01) after exogenous estrogen supplementation.

**Table 3:** Body weight of rats in each group

	Weight(g)			
**Week**	Sham	OVX	ALN	YSGSF
0	288.8 ± 5.0	286.7 ± 14.9	277.7 ± 8.8	276.0 ± 19.2
1	301.3 ± 7.9	306.7 ± 17.2	306.7 ± 4.9	293.3 ± 16.5
2	303.3 ± 12.0	338.7 ± 14.9^##^	312.3 ± 13.7^*^	307.5 ± 18.3^**^
3	316.2 ± 13.7	355.5 ± 26.1^#^	318.5 ± 12.9^*^	315.3 ± 23.9^*^
4	333.3 ± 27.4	376.2 ± 25.8^#^	336.3 ± 15.3^*^	325.7 ± 25.9^**^
5	334.3 ± 13.7	381.5 ± 18.7^##^	336.8 ± 12.9^**^	324.0 ± 25.1^**^
6	332.3 ± 13.4	378.0 ± 31.9^##^	345.2 ± 12.5^*^	337.3 ± 16.0^*^
7	340.2 ± 11.9	399.2 ± 28.8^##^	358.2 ± 9.8^**^	350.8 ± 12.6^**^
8	346.2 ± 12.1	413.0 ± 26.6^##^	369.2 ± 14.0^**^	356.8 ± 18.6^**^
9	354.5 ± 12.5	423.0 ± 26.1^##^	382.2 ± 16.3^*^	370.3 ± 22.0^**^
10	361.0 ± 15.3	429.7 ± 30.7^##^	395.7 ± 20.3^*ns*^	381.3 ± 32.9^*^
11	364.3 ± 11.6	442.0 ± 27.7^##^	400.7 ± 14.9^*^	387.7 ± 37.8^**^
12	362.8 ± 11.7	459.8 ± 30.7^##^	413.0 ± 12.7^*#^	399.5 ± 36.5^**^

Compared to the Sham group, ^*#*^*p* < 0.05, ^*##*^*p* < 0.01; Compared to the OVX group, ^*^p < 0.05, ^**^p < 0.01; ^ns^*p*>0.05. (*n* = 6)

### YSGSF improves bone metabolism in OVX rats

To evaluate the regulatory effect of YSGSF on bone metabolism in OVX rats, enzyme-linked ELISA was used to measure the levels of serum bone metabolism markers in each group. As shown in Fig. 1C and D, compared with the Sham group, the serum level of PINP in the OVX group was significantly decreased (*P* < 0.01), while the serum level of β-CTX was significantly higher than that in the Sham group (*P* < 0.01). These results indicate that bone formation was significantly inhibited and bone resorption activity was remarkably enhanced after OVX surgery. After intervention with ALN and YSGSF, the serum PINP levels in both the ALN group and YSGSF group were significantly higher than those in the OVX group (*P* < 0.01), and the serum β-CTX levels were significantly lower than those in the OVX group (*P* < 0.01); there was no significant difference between the ALN group and YSGSF group.

Taken together, analysis of serum bone metabolism markers demonstrates that YSGSF exerts a bidirectional regulatory function on bone metabolism—it effectively ameliorates OVX-induced bone metabolic disorder by promoting bone formation and inhibiting bone resorption.

### YSGSF improves osteoporosis in OVX rats

To confirm the inhibitory effect of YSGSF on PMOP by regulating bone metabolism, this study further evaluated the bone tissue morphology of the distal femur in rats from each group via HE staining. As shown in Fig. 1E, the Sham group exhibited dense, continuous, and uniformly distributed trabecular bone structures. In contrast, the OVX group showed significant abnormalities in bone structure, characterized by a marked reduction in the number of trabecular bones, sparse distribution, impaired structural integrity, and adipocyte infiltration in the bone marrow cavity—these changes indicate the presence of pathological alterations associated with PMOP. After drug intervention, both the ALN group and YSGSF group displayed improvements in bone structure, including an increased number of trabecular bones, restored structural continuity, and enhanced inter-trabecular connections.

Morphological analysis based on HE staining results demonstrated that YSGSF can effectively reverse the OVX-induced pathological changes of osteoporosis and improve the morphological structure of bone tissue by regulating bone metabolism.

### YSGSF improves bone tissue microstructure in OVX rats

**Fig. 2:**
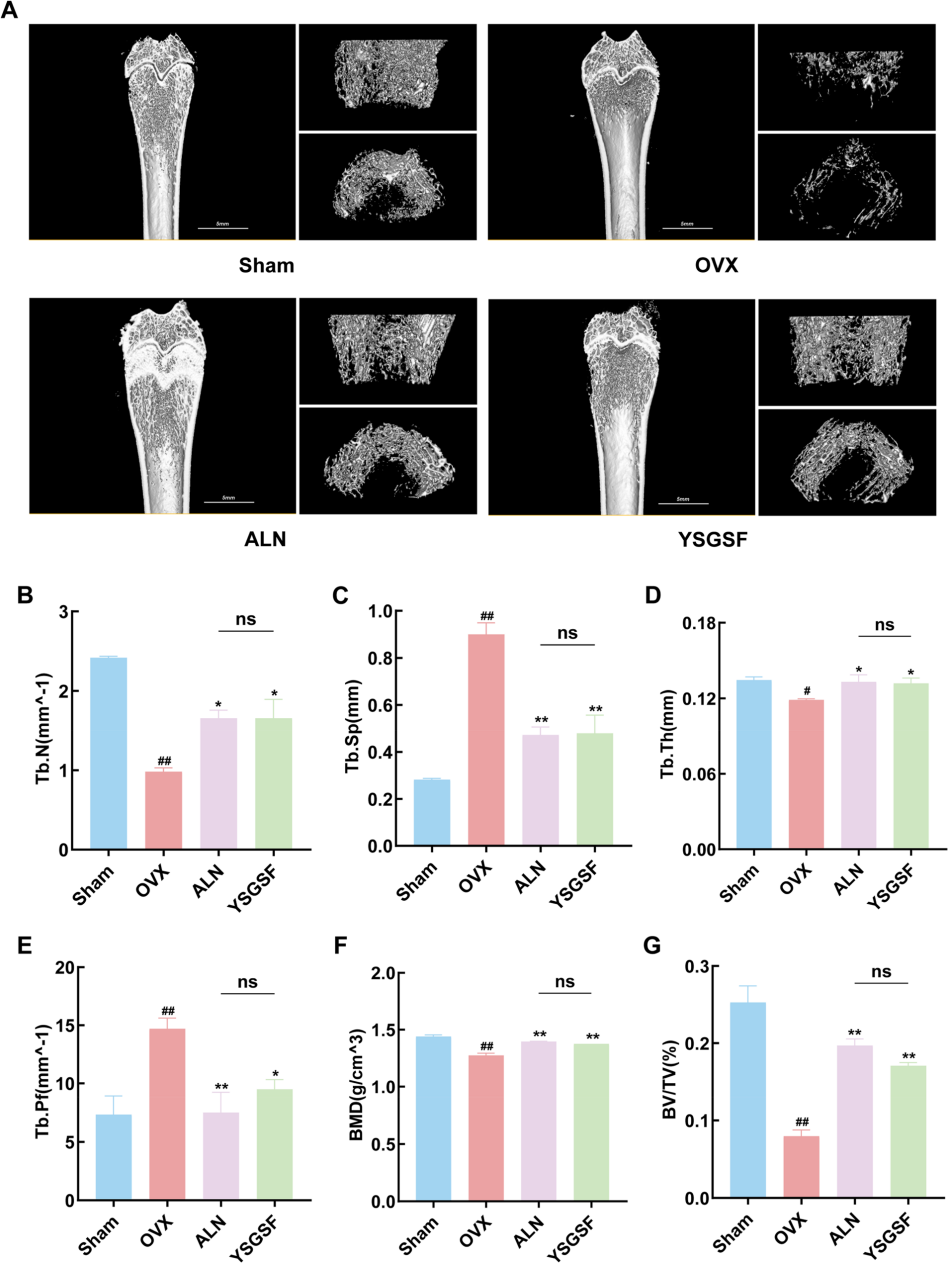
YSGSF improves the bone tissue microstructure in OVX rats. **A** Micro-CT of the distal femur in each group. **B-E** Values of Tb.N, Tp.Sp, Tb.Th, and Tb.Pf in each group. **(F**,** G)** Values of BMD and BV/TV in each group. (*n* = 3). Compared to the Sham group, ^*#*^*p* < 0.05, ^*##*^*p* < 0.01; Compared to the OVX group, ^*^p < 0.05, ^**^p < 0.01; ^*ns*^*p*>0.05

To further evaluate the effect of YSGSF on bone structure, this study employed Micro-CT technology for high-resolution 3D imaging and systematic quantitative analysis of the femurs in rats from each group. As shown in Fig. 2A, the 3D reconstructed images of the distal femur clearly revealed differences in bone structure among the groups. Compared with the OVX group, both the ALN group and YSGSF group exhibited a more complete and continuous 3D trabecular structure in the distal femur, with higher trabecular network density, significant recovery in bone density and bone mass, and these parameters were close to the levels observed in the Sham group.

Further quantitative analysis of bone microstructure parameters (Fig. 2B–E) showed that, compared with the OVX group, the trabecular number (Tb.N) and trabecular thickness (Tb.Th) in the ALN group and YSGSF group were significantly increased, while the trabecular separation (Tb.Sp) and trabecular pattern factor (Tb.Pf) were notably decreased. These changes indicate that both ALN and YSGSF treatments effectively improved the trabecular structure of OVX rats and reduced the porosity and pore volume of bone tissue.

In addition, as shown in Fig. 2F and G, the bone mineral density (BMD) and bone volume fraction (BV/TV) in the ALN group and YSGSF group were significantly higher than those in the OVX group. The above results further demonstrate that YSGSF can effectively promote mineral deposition in the bone matrix, improve the overall quality of bone tissue, and further verify its positive role in maintaining bone structure health; moreover, its therapeutic effect is comparable to that of ALN.

### RNA-Seq analysis of YSGSF in treating OVX rats

The aforementioned studies systematically demonstrated that YSGSF exerts significant osteoprotective effects in the OVX-induced PMOP rat model. However, the specific molecular mechanisms underlying these beneficial effects of YSGSF remain to be further elucidated. To this end, this study employed RNA-seq technology to conduct a comparative analysis of the whole-genome expression profiles of bone tissues between the YSGSF group and the OVX group, aiming to systematically identify the potential molecular targets and signaling pathways through which YSGSF regulates bone metabolism at the transcriptomic level.

**Fig. 3:**
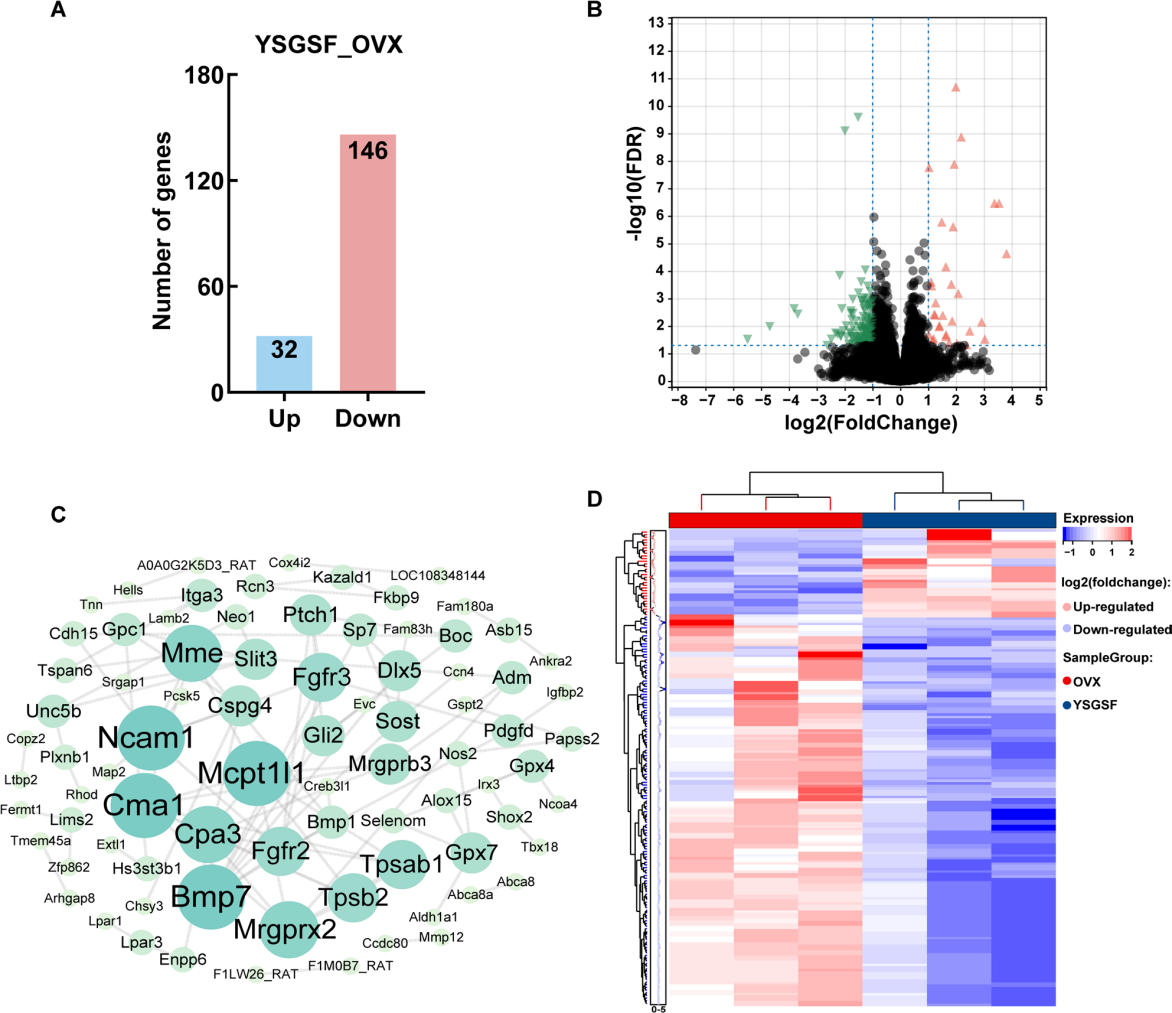
RNA-seq of bone tissue from the YSGSF and OVX groups. **A** The number of differentially expressed genes (DEGs) in the YSGSF group compared to the OVX group. **B** Volcano plot of DEGs, where the red arrows indicate upregulated genes and the green arrows indicate downregulated genes. **C** DEGs PPI network. **D** Heatmap of the expression matrix for the 178 DEGs

The sequencing analysis results showed (Fig. 3A) that with the OVX group as the control, there were 178 significantly differentially expressed genes (DEGs) in the bone tissues of the YSGSF group. Among these DEGs, 32 genes were significantly upregulated, and 146 genes were significantly downregulated. The specific expression fold changes of the DEGs are provided in Table S1, and the expression trends of the DEGs are shown in Fig. 3B. By constructing a protein-protein interaction (PPI) network for all DEGs (Fig. 3C), a gene interaction network with 196 connection edges was formed; among them, genes represented by larger circles had higher connectivity with other genes, indicating that most DEGs have extensive mutual interactions. The matrix heatmap showing the expression trends of all DEGs is presented in Fig. 3D.

**Fig. 4:**
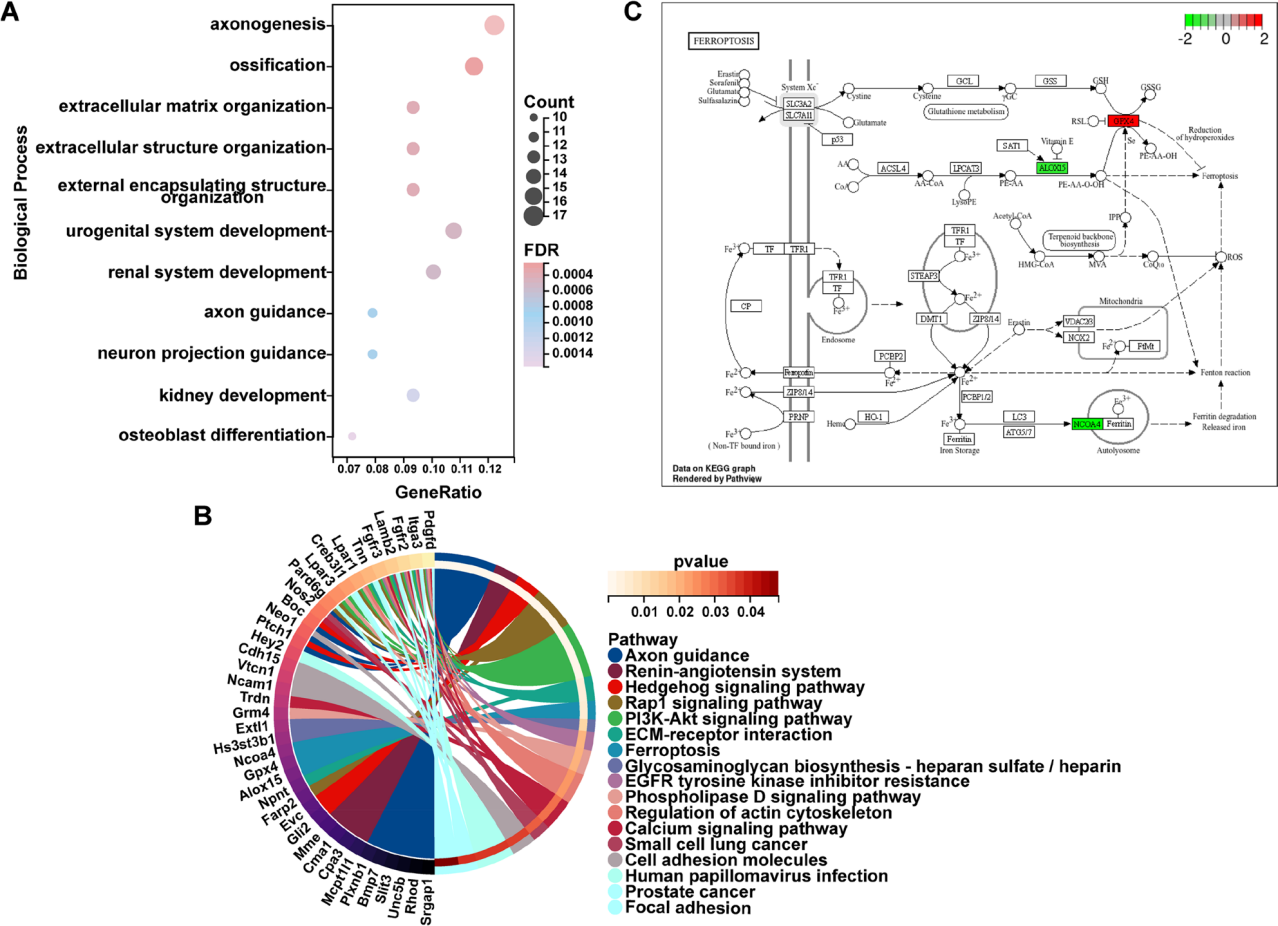
GO and KEGG enrichment analysis of the 178 DEGs. **A** Biological Process terms from the GO enrichment analysis results. **B** KEGG enrichment analysis. **C** Enrichment of DEGs in the Ferroptosis pathway, where green indicates downregulated genes and red indicates upregulated genes

Further GO (Gene Ontology) and KEGG (Kyoto Encyclopedia of Genes and Genomes) enrichment analyses were performed on all DEGs. The GO enrichment results indicated that the biological processes (BP) enriched by DEGs were mainly concentrated in processes such as “ossification”, “osteoblast differentiation” and “skeletal development”. This not only explains the previously observed therapeutic effect of YSGSF on regulating bone metabolism, but also suggests that the therapeutic effect of YSGSF on PMOP may be closely associated with regulating osteoblast differentiation and promoting bone formation. All GO enrichment results are provided in Table S2. The main KEGG enrichment results (Fig. 4B) included “Rap1 signaling pathway”, “PI3K-Akt signaling pathway” and “ferroptosis”, suggesting that YSGSF may interfere with mechanisms such as osteoblast differentiation by regulating signaling axes including ferroptosis. The key DEGs enriched in the ferroptosis signaling pathway include ALOX15, GPX4 and NCOA4. As shown in Fig. 4C, compared with the OVX group, the expression levels of NCOA4 and ALOX15 in the bone tissues of rats treated with YSGSF exhibited a downregulated trend, while the expression level of GPX4 showed an upregulated trend.

In summary, the differential gene analysis of bone tissues between the OVX group and the YSGSF group indicates that YSGSF improves osteoporosis in OVX rats through multiple mechanisms and multiple targets. As mentioned earlier, ferroptosis is closely associated with the occurrence and development of PMOP; this suggests that YSGSF may treat PMOP by regulating the ferroptosis signaling pathway to interfere with osteoblast differentiation and promote bone formation. Based on this, subsequent experiments in this study conducted relevant detections focusing on key factors such as ferroptosis and iron metabolism, and also paid attention to some ferroptosis-related products.

### YSGSF regulates iron metabolism in OVX rats

**Fig. 5:**
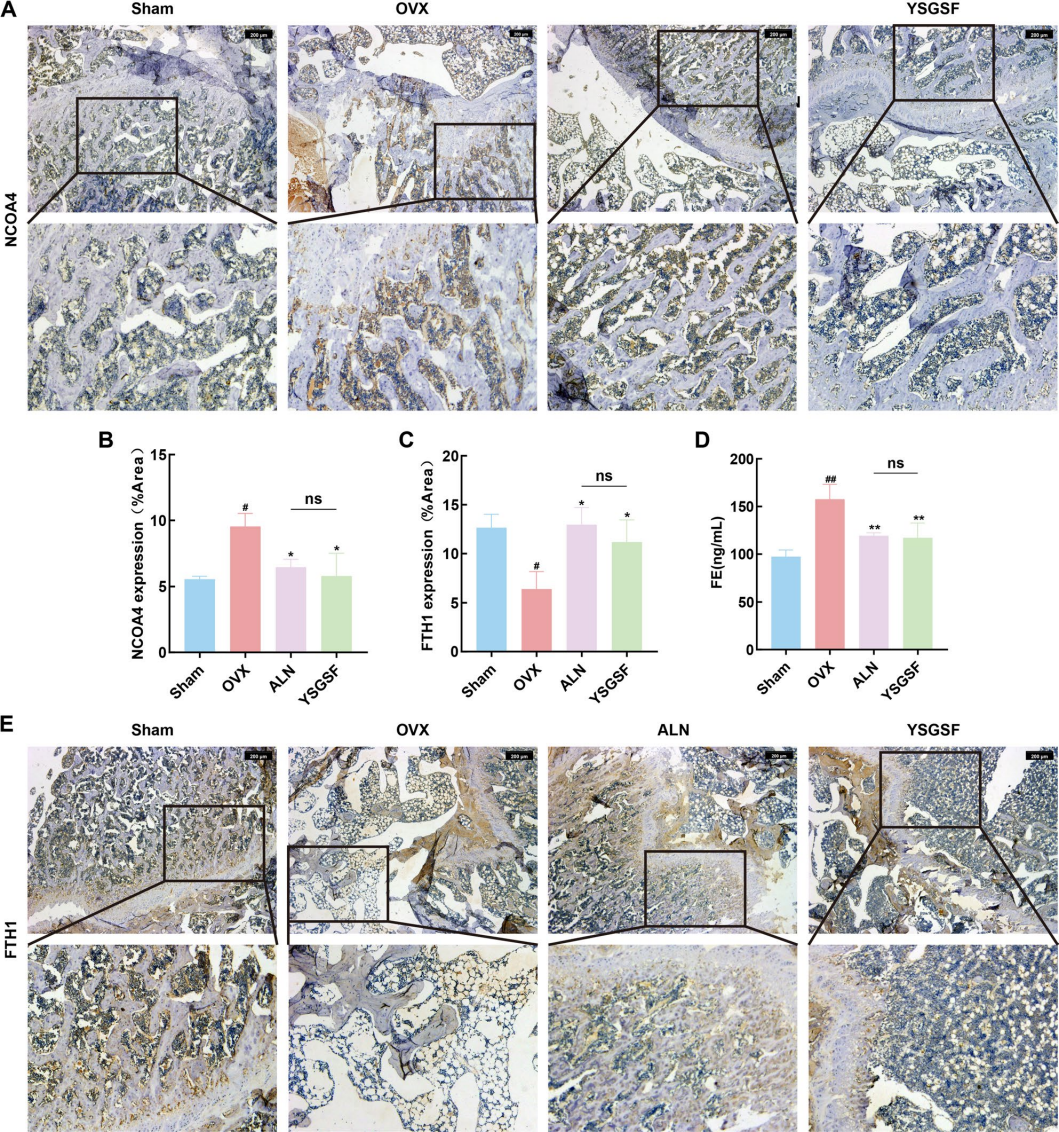
YSGSF regulates FE metabolism in OVX rats. **A** IHC staining of NCOA4 in the distal bone tissue of rats from each group. **B** The positive expression area of NCOA4 in each group. **C** The positive expression area of FTH1 in each group. **D** Serum FE levels from each group. **E** IHC staining of FTH1 in the distal bone tissue of rats from each group. Compared to the Sham group, ^*#*^*p* < 0.05, ^*##*^*p* < 0.01; Compared to the OVX group, ^*^p < 0.05, ^**^p < 0.01; ^*ns*^*p*>0.05

Ferroptosis is an iron-dependent lipid peroxidation-induced damage, and iron ion metabolism disorder acts as a catalyst for ferroptosis. To clarify the specific mechanism by which YSGSF regulates iron ion metabolism, IHC detection of iron metabolism-related factors was performed on bone tissues in this study. Results are shown in Fig. 5A-B: compared with the other three groups, the positive expression area of NCOA4 in the femoral tissues of rats in the OVX group was significantly increased (*P* < 0.05), and this high expression was mainly concentrated in trabecular bone and osteoblasts.

Meanwhile, the expression of FTH1 in bone tissues and serum iron ion levels were detected. As shown in Fig. 5C-E, the expression of FTH1 in OVX bone tissues was significantly decreased (*P* < 0.05), while serum iron ion levels were significantly higher than those in the Sham group (*P* < 0.01). Combined with the above results, compared with the Sham group, rats in the OVX group exhibited obvious iron metabolism disorder; the upregulated expression of NCOA4 may have promoted the release of a large amount of free iron into the systemic circulation.

On the other hand, compared with rats in the OVX group, the high expression of NCOA4 in bone tissues of rats in the treatment groups (ALN and YSGSF groups) was significantly reduced, the downregulation of FTH1 expression was alleviated, and the serum FE levels in the YSGSF group also tended to be normal. These findings indicate that YSGSF may improve iron ion metabolism and reduce iron accumulation in OVX rats through the NCOA4/FTH1 signaling axis.

### YSGSF inhibits ferroptosis in OVX rats

**Fig. 6:**
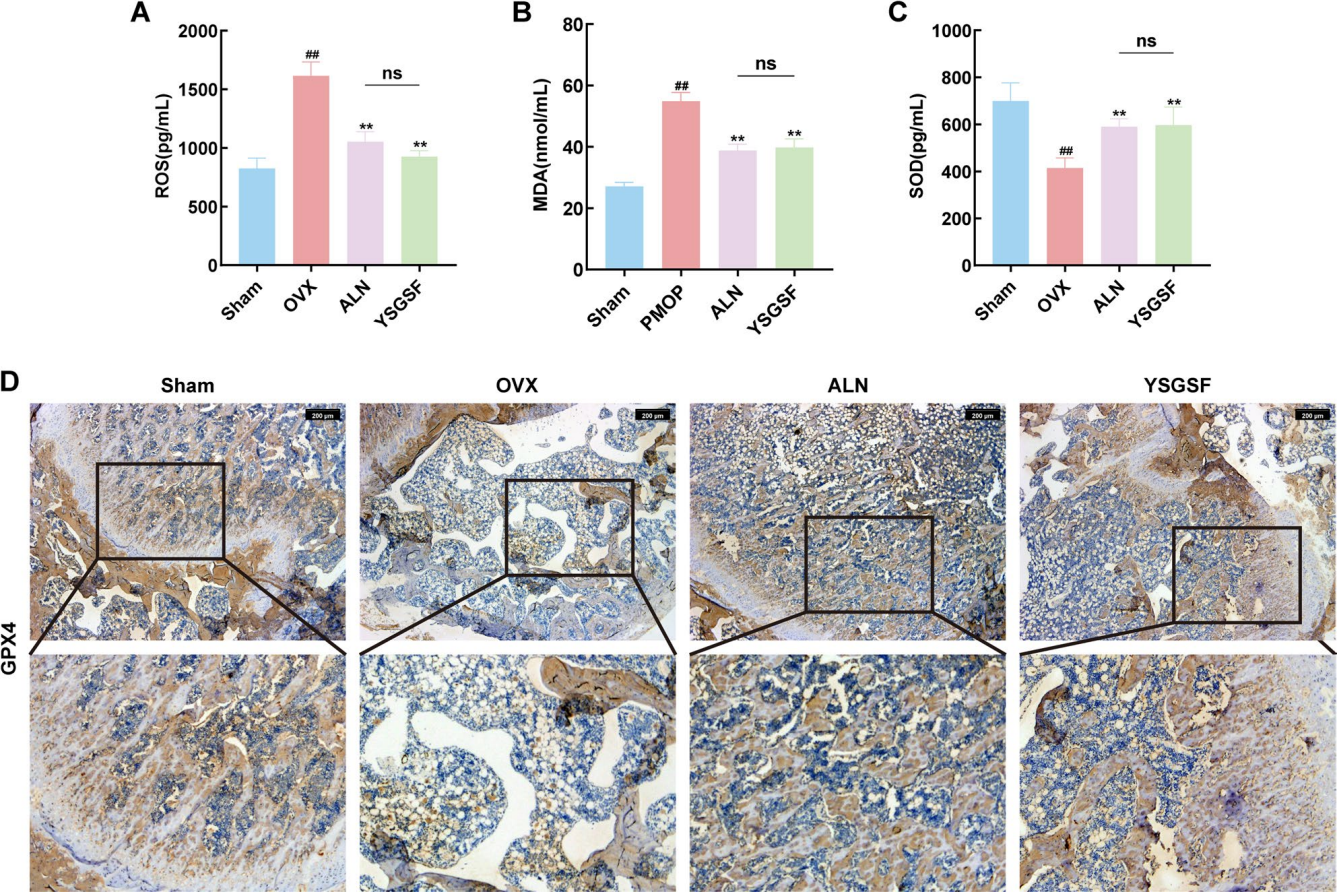
YSGSF alleviates oxidative damage in OVX rats. **A** Serum ROS levels in each group. **B** Serum MDA levels in each group. **C** Serum SOD levels in each group. **D** IHC staining of GPX4 in the distal bone tissue of rats from each group. Compared to the Sham group, ^*#*^*p* < 0.05, ^*##*^*p* < 0.01; Compared to the OVX group, ^*^p < 0.05, ^**^p < 0.01; ^*ns*^*p*>0.05

Iron metabolism disorder provides substrates for ferroptosis and serves as a prerequisite for this process. Based on the results of the previous section, this study further detected ferroptosis-related products. As shown in Fig. 6A-C, compared with the Sham group, the serum levels of ROS and MDA in the OVX group were increased, while SOD levels were significantly decreased (*P* < 0.01). These findings indicate that the accumulation of serum iron ions further induces the production of lipid peroxides.

**Fig. 7:**
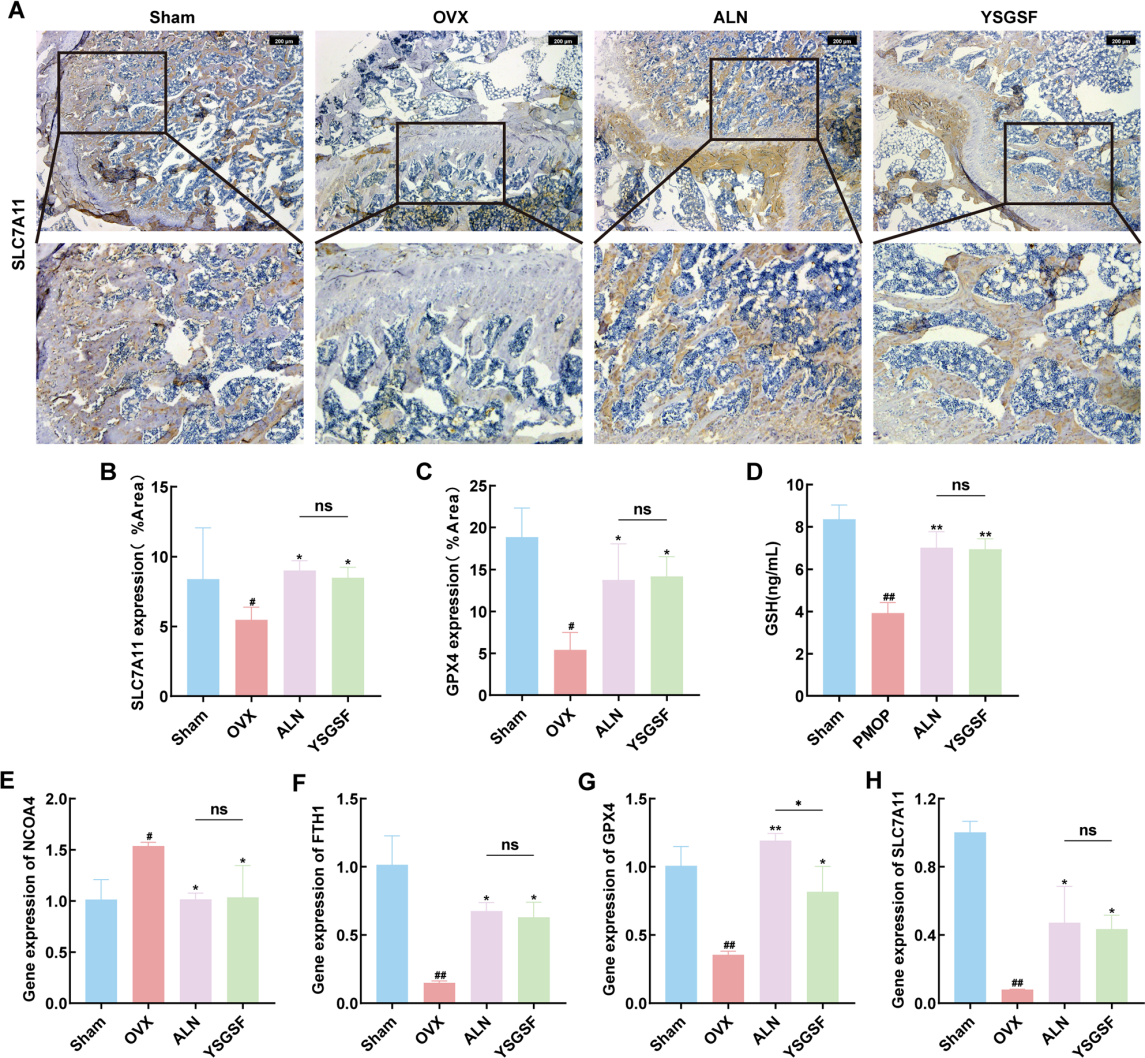
YSGSF alleviates ferroptosis in OVX rats. **A** IHC staining of SLC7A11 in the distal bone tissue of rats from each group. **B** The positive expression area of SLC7A11 in each group. **C** The positive expression area of GPX4 in each group. **D** Serum GSH levels in each group. **E** NCOA4 mRNA expression levels in each group. **F** FTH1 mRNA expression levels in each group. **G** GPX4 mRNA expression levels in each group. **H** SLC7A11 mRNA expression levels in each group. Compared to the Sham group, ^*#*^*p* < 0.05, ^*##*^*p* < 0.01; Compared to the OVX group, ^*^p < 0.05, ^**^p < 0.01; ^*ns*^*p*>0.05

IHC staining of rat femoral tissues revealed (Figs. 6D and 7A-C) that compared with the Sham group, the positive expression areas of ferroptosis key factors (GPX4 and SLC7A11) in the bone tissues of the OVX group were significantly reduced (*P* < 0.05), accompanied by a decrease in GSH levels (Fig. 7D). These results confirm that during the progression of PMOP in OVX rats, the body’s antioxidant capacity is weakened and antioxidant-related enzymes are reduced, leading to iron-dependent lipid peroxidation.

On the other hand, compared with the OVX group, after intervention with ALN and YSGSF, the positive expressions of GPX4 and SLC7A11 in the bone tissues of the two treatment groups were restored, with positive staining mainly concentrated in the trabecular bone. Meanwhile, the serum levels of ROS and MDA in the two groups decreased, confirming that YSGSF treatment alleviates oxidative damage and restores antioxidant factors, as evidenced by the increased levels of SOD and GSH.

The above IHC staining results were further verified at the gene level by qRT-PCR, and the mRNA expression levels were consistent with the aforementioned findings (Fig. 7E-H). Specifically, the mRNA expression trends of NCOA4 and GPX4 in the YSGSF group (compared with the OVX group) were consistent with the results of RNA-seq enrichment.

Therefore, integrating the above results, we propose that the main mechanism by which YSGSF exerts its therapeutic effect on PMOP may involve regulating the NCOA4/GPX4 signaling pathway to improve iron metabolism, further inhibit lipid peroxidation-induced ferroptosis, and ultimately promote bone formation while reducing bone loss.

### YSGSF alleviates Erastin-induced ferroptosis in ROS17/28 osteoblasts

**Fig. 8:**
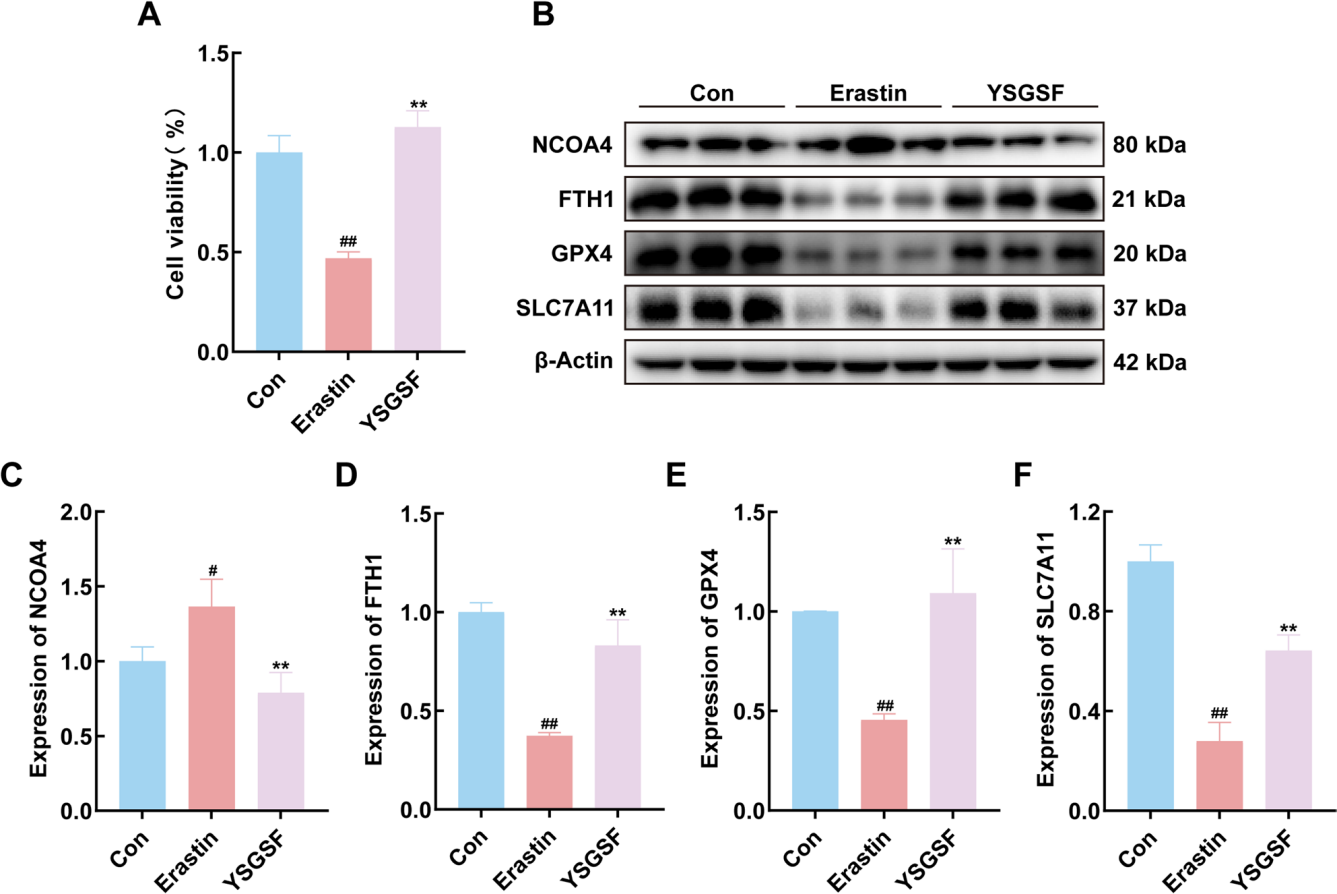
YSGSF ameliorates Erastin-induced ferroptosis in ROS17/28 cells. **A** Cell viability of ROS17/28 cells in each group. **B** The bands of NCOA4, FTH1, GPX4, SLC7A11, and β-Actin in each group. **C** Quantitative analysis of NCOA4 protein (**D**) Quantitative analysis of FTH1 protein (**E**) Quantitative analysis of GPX4 protein (**F**) Quantitative analysis of SLC7A11 protein. Compared to the Con group, ^*#*^*p* < 0.05, ^*##*^*p* < 0.01; Compared to the Erastin group, ^*^p < 0.05, ^**^p < 0.01; ^*ns*^*p*>0.05

To investigate the regulatory effect of YSGSF on Erastin-induced ferroptosis, the experiment was divided into control group (Con), Erastin-treated group, and YSGSF intervention group. The cell viability assay results shown in Fig. 8A demonstrated that the Con group maintained high cell viability. After Erastin treatment, cell viability significantly decreased (*P* < 0.01). However, following YSGSF intervention, cell viability markedly recovered compared to the Erastin group (*P* < 0.01), indicating that YSGSF could alleviate Erastin-induced cell death.

To elucidate the regulatory mechanism of YSGSF on ferroptosis, the expression levels of key ferroptosis-related proteins (NCOA4, FTH1, GPX4, and SLC7A11) were detected by Western blot (Fig. 8B), followed by quantitative analysis of the bands. The results showed that compared with the Con group, the expression of NCOA4 was significantly increased in the Erastin group (*P* < 0.05, Fig. 8C). After YSGSF intervention, the expression of NCOA4 was significantly downregulated compared with the Erastin group (*P* < 0.01). Meanwhile, Erastin treatment resulted in a significant decrease in FTH1 expression compared to the Con group (*P* < 0.01, Fig. 8D); YSGSF intervention markedly upregulated FTH1 expression (*P* < 0.01 vs. Erastin group). Compared with the Con group, the Erastin group showed significantly reduced expression of GPX4 and SLC7A11 (*P* < 0.01, Fig. 8E, F); after YSGSF intervention, the protein expression levels were significantly restored compared to the Erastin group (*P* < 0.01). In summary, Erastin can induce osteoblast ferroptosis by upregulating NCOA4 and downregulating the expression of SLC7A11/GPX4/FTH1, while YSGSF is capable of reversing the abnormal expression of ferroptosis-related proteins, thereby inhibiting Erastin-mediated osteoblast ferroptosis.

## Discussion

This study established a postmenopausal osteoporosis (PMOP) rat model through ovariectomy (OVX) and administered Yishen Gugu Formula (YSGSF) as an intervention. A series of in vivo and in vitro experiments indirectly elucidated the therapeutic effects of YSGSF on PMOP and explored its molecular mechanism of inhibiting ferroptosis by regulating the NCOA4/GPX4 signaling pathway.

As an extension and expansion of previous research, the findings of this study not only provide new theoretical evidence for the osteoprotective effect of YSGSF but also offer a novel strategy for the treatment of PMOP.

In recent years, in the field of PMOP research, scholars at home and abroad have focused on in-depth exploration of the important role of ferroptosis in bone metabolism imbalance. Numerous studies have indicated that the sharp decline in estrogen levels in women after menopause is a core characteristic of the pathogenesis of PMOP, and this decline directly disrupts the dynamic balance between bone formation and bone resorption. This is mainly reflected in the fact that within 3–5 years after menopause, the incidence of PMOP gradually increases from 30% to 50%, which is 3–5 times that of men of the same age [38, 39]. The main reason is that estrogen, as an important antioxidant in the body, its reduction leads to a decrease in the body’s antioxidant capacity [40, 41]. The main pathological features include bone loss, increased oxidative stress, and iron metabolism disorders, which are consistent with the results of this study.

Reactive oxygen species (ROS) acts as a hub between cellular ferroptosis and iron metabolism disorders. The core process of ferroptosis involves iron-dependent lipid peroxidation reactions; excessive free iron promotes the massive production of lipid peroxides such as ROS and malondialdehyde (MDA), providing raw materials for the occurrence of ferroptosis [8]. Ouyang et al. pointed out that the expression of bone resorption factors is positively correlated with the level of oxidative stress, and the increase in ROS promotes osteoclast differentiation [42]. β-C-terminal telopeptide of type I collagen (β-CTX) is a clinically effective diagnostic indicator reflecting the rate of bone resorption, and is also considered a more suitable diagnostic marker for elderly women [43]. Some studies have noted that the increase in peroxides such as ROS and MDA is often accompanied by an elevation in β-CTX [43].

In osteoblasts, reducing ROS production and restoring the expression of antioxidant factors (e.g., superoxide dismutase [SOD], glutathione [GSH], glutathione peroxidase [GPX]) can significantly alleviate cell apoptosis induced by oxidative stress, while also significantly promoting the expression of osteogenic indicators such as procollagen type I N-terminal propeptide (PINP) and runt-related transcription factor 2 (Runx2) [44]. These aforementioned studies and the present study have confirmed at different levels that the weakening of the body’s antioxidant capacity is a direct factor leading to increased ROS production and subsequent impairment of bone metabolism. In PMOP prevention and treatment research, regulating iron metabolism is crucial for further inhibiting ferroptosis and restoring bone homeostasis [8, 31, 32].

Notably, the inhibition of iron ion transport exacerbates oxidative damage and induces ferroptosis [45]. Studies have shown that ferritin heavy chain 1 (FTH1), as the heavy chain of ferritin, exerts an iron storage function by chelating labile iron ions, thereby reducing ROS production catalyzed by iron ions in the Fenton reaction. Nuclear receptor coactivator 4 (NCOA4)-mediated ferritin autophagy promotes the degradation of FTH1 and the release of large amounts of free iron (FE), which amplifies oxidative damage and ultimately triggers ferroptosis [29]. Downregulated FTH1 expression further impairs the cell’s iron storage capacity, aggravates cell dysfunction, and has been reported to be positively correlated with decreased bone mineral density (BMD) [46]. During osteogenic differentiation, overexpression of NCOA4 increases the deposition of iron ions and lipid peroxides, leading to ferroptosis of bone marrow mesenchymal stem cells (BMSCs) [47]. Ni et al. indicated that NCOA4 overexpression is significantly associated with osteoclast differentiation; the process of NCOA4/FTH1 autophagosome degrading ferritin activates receptor activator of nuclear factor-κB ligand (RANKL), suggesting an interaction between iron metabolism disorders and molecules related to excessive bone resorption [48].

Thus, iron homeostasis imbalance mediated by the NCOA4/FTH1 axis is closely associated with ferroptosis-driven bone degeneration in the pathogenesis and progression of PMOP. In the present study, our focus on the NCOA4/FTH1 signaling pathway and key ferroptosis factors (GPX4 and SLC7A11) confirmed, from another perspective, that the inhibition of ferroptosis in the pathological progression of PMOP is related to the repair of the antioxidant system.

With the in-depth study of the pathogenesis of PMOP, TCM has shown broad application prospects in the prevention and treatment of PMOP due to its unique theoretical system and rich practical experience [49, 50, 51). Scholars at home and abroad have conducted extensive explorations on the mechanisms underlying TCM treatment for PMOP [52–54]. Building on previous research, this study further verified that YSGSF may inhibit ferroptosis by regulating the NCOA4/GPX4 signaling pathway, thereby providing a new option for TCM-based PMOP treatment.

Compared with previous studies, this research further explored the critical role of ferroptosis in bone metabolism during the progression of PMOP, and elucidated the scientific connotation of the TCM theory “the kidney governs the bones” through modern molecular biology techniques. However, this study still has certain limitations. The current findings indicate that ferroptosis is a highly relevant mechanism, but establishing its core causal relationship still requires in-depth validation in future studies. These experiments will be prioritized in subsequent work to provide more conclusive evidence. Meanwhile, the study did not identify the main components of YSGSF that mediate its specific mechanisms of action, nor did it address questions such as how these active components exert synergistic effects. This indicates that in future research, we can further explore the molecular mechanisms of YSGSF in greater depth by integrating techniques such as mass spectrometry analysis and pharmacokinetics.

## Supplementary Information

Supplementary Material 1.

Supplementary Material 2.

Supplementary Material 3.

## Data Availability

The data sets generated and analyzed during this study can not be disclosed temporarily because the relevant research projects are still in progress, but can be obtained from the corresponding authors upon reasonable request.

## Abbreviations

ALN: Alendronate sodium
BMD: Bone mineral density
BSA: Bovine Serum Albumin
BV/TV: Bone volume fraction
β-CTX: β-C-terminal telopeptide of type I collagen
ELISA: Enzyme-Linked Immunosorbent Assay
EXD: Erxian Decoction
FTH1: Ferritin heavy chain 1
FTL: Ferritin light chain
FLJC: Fufang Lurong Jiangu capsule
GSH: Glutathione
GPX4: Glutathione peroxidase 4
GO: Gene Ontology
HE: Hematoxylin-Eosin
IHC: Immunohistochemistry
KEGG: Kyoto Encyclopedia of Genes and Genomes
MDA: Malondialdehyde
NCOA4: Nuclear receptor coactivator 4
OVX: Ovariectomized
PPI: Protein-protein interaction
PMOP: Postmenopausal osteoporosis
PINP: Procollagen type I N-terminal propeptide
qRT-PCR: Quantitative real-time polymerase chain reaction
QEP: Qing`e Pill
ROS: Reactive oxygen species
SLC7A11: Solute carrier family 7 member 11
SD: Sprague-Dawley
SOD: Superoxide dismutase
SPF: Specific pathogen-free
TCM: Traditional Chinese Medicine
Tb.N: Trabecular number
Tb.Pf: Trabecular pattern factor
Tb.Sp: Trabecular separation
Tb.Th: Trabecular thickness
RNA-seq: RNA sequencing
ZGW: Zuogui Pill
YSGSF: Yishen Gushu Formula
