# Supplementary material for: Yishen Gushu formula exerts osteoprotective effects in OVX-Induced PMOP rats: role of ferroptosis Inhibition and iron metabolism correction
Source: Hereditas. 2026 Feb 4;163:34. doi: 10.1186/s41065-026-00642-5 (PMC12924249; doi:10.1186/s41065-026-00642-5)
Supplement: Supplementary file 3 — Supplementary Material 3. [file 41065_2026_642_MOESM3_ESM.docx]

**1.The effect of Erastin on ROS17/28 cells.**

Based on CCK-8 assay results, Erastin demonstrated a clear concentration-dependent effect on the viability of ROS17/28 osteoblasts (as shown in Figure 1A). Within the concentration range of 0-5 µM (0.1, 0.5, 1, 5 µM), no significant changes in cell viability were observed compared to the solvent control group (0 µM) (P > 0.05), indicating that low concentrations of Erastin did not exhibit obvious inhibitory effects on cell activity. When the concentration increased to 10 µM and 20 µM, cell viability significantly decreased (P < 0.0001), with the 20 µM group showing the most pronounced inhibitory effect. As shown in Figure 1B, the dose-response curve fitting results demonstrated that the IC50 value of Erastin was 2.15 µM, and this concentration was subsequently used as the intervention dose for ROS17/28 osteoblasts.


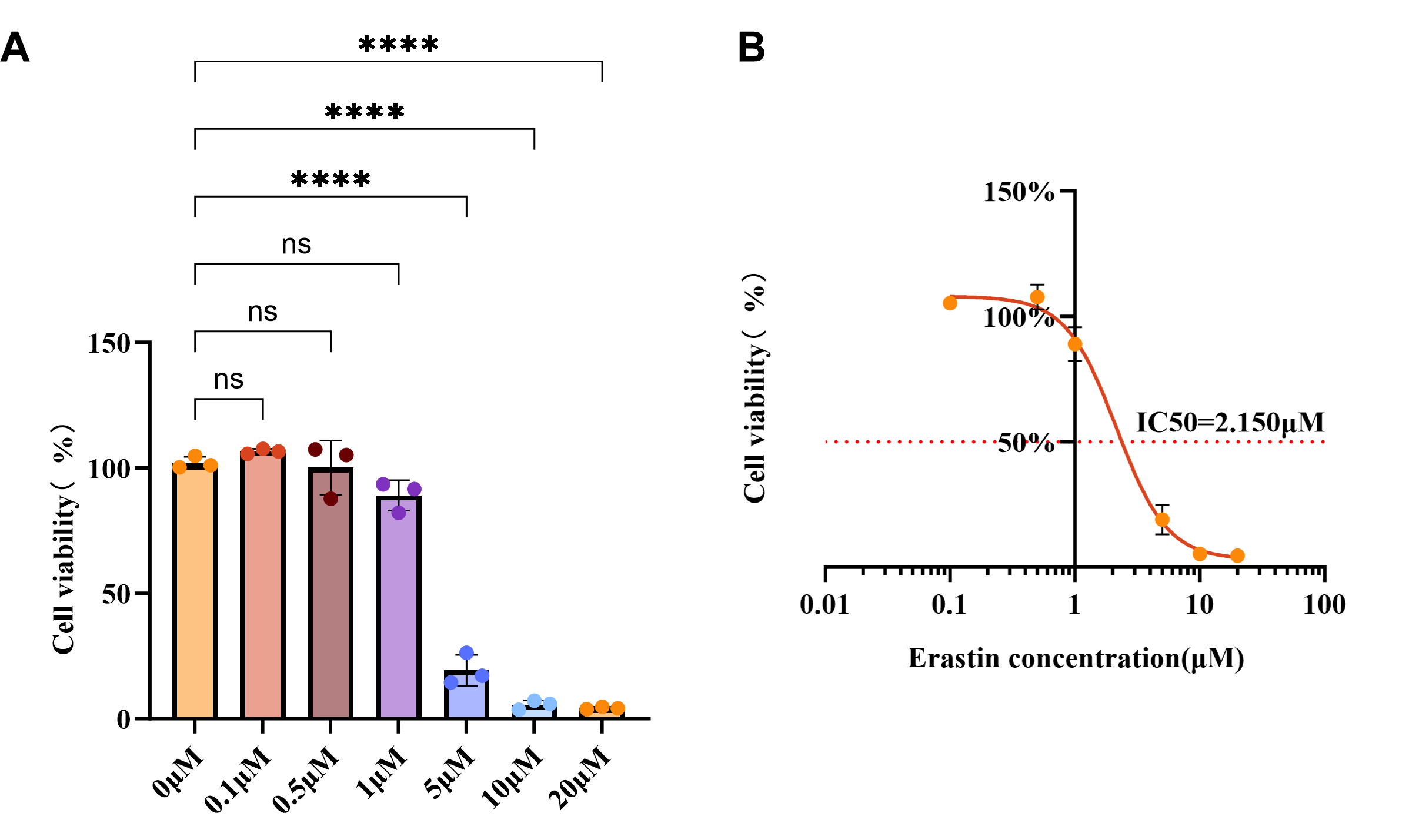


**Figure 1 Effect of Erastin at different concentrations on the proliferation of ROS17/28 osteoblasts.** Compared with the 0µM, * P < 0.05, ** P < 0.01, *** P < 0.001, **** P < 0.0001 ; ns P > 0.05.

**2. Screening of the optimal concentration of YSGSF-containing serum for intervention in ROS17/28 cells.**

Based on the CCK-8 assay results, the effects of YSGSF-containing serum at different concentrations on the viability of ROS17/28 osteoblasts are shown in Figure 2. Compared with the Control group, the cell viability in the Blank serum group was 100% with no statistically significant difference, indicating that the rat-derived serum matrix itself had no significant effect on cell activity. Figure 2 demonstrates that compared with the Control group, the 40% YSGSF-containing serum group showed a significant decrease in cell viability to 50.00±5.00% (P 0.05), but demonstrating a trend of activity recovery. The 5% YSGSF group exhibited restored viability to 90.00±2.00%, while the 2.5% YSGSF group showed a significant increase to 85±6.00% (P > 0.05). The results indicate that 5% YSGSF drug-containing serum may have a proliferative-promoting effect as the optimal intervention concentration, and therefore 5% YSGSF serum was selected as the intervention dose for ROS17/28 osteoblasts in subsequent experiments.


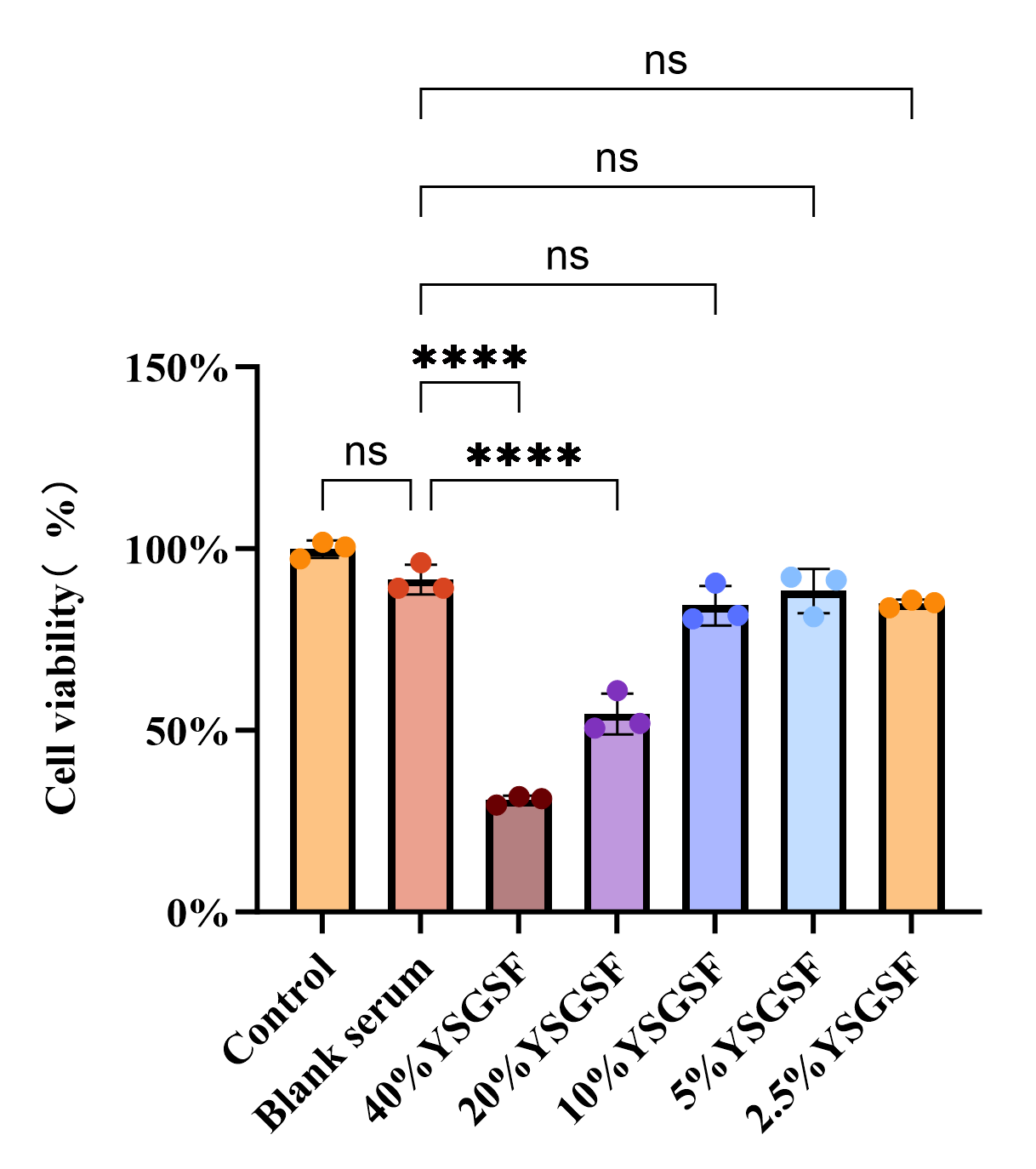


# **Figure 2 Effects of YSGSF-containing serum at different concentrations on ROS17/28 osteoblasts.** Compared with the Blank serum, * P < 0.05, ** P < 0.01, *** P < 0.001, **** P < 0.0001 ; ns P > 0.05.
